# The aberrant systemic-pulmonary artery communication: three-dimensional image simulation

**DOI:** 10.1186/s13019-019-0855-5

**Published:** 2019-02-11

**Authors:** Chun-Lin Kuo, Kuan-Hsun Lin, Kai-Hsiung Ko, Tsai-Wang Huang

**Affiliations:** 1 Tri-Service General Hospital, National Defense Medical Center, Taipei, Taiwan, Republic of China; 2 Department of Surgery, Division of Thoracic Surgery, Tri-Service General Hospital, National Defense Medical Center, 325, Section 2, Cheng-Kung Road, Taipei, 114 Taiwan, Republic of China; 3 Department of Radiology, Tri-Service General Hospital, National Defense Medical Center, Taipei, Taiwan, Republic of China

**Keywords:** Hemoptysis, Anomalous arterial supply, 3-D image simulation

## Abstract

**Background:**

Congenital arterial abnormalities are unusual causes of hemoptysis in elder patients. Appropriate image examination and recognition of the variant vessels are crucial in achieving precise diagnosis and successful treatment.

**Case presentation:**

We report a case of 70-year-old female presenting with hemoptysis. Computed tomography angiography showed hypervascular lesions in the lingula of the left lung abutting the pericardium. Three-dimensional reconstruction image revealed an aberrant systemic arterial supply communicating with the left pulmonary artery and co-supplying the pulmonary parenchyma of the left upper. Single-port video-assisted thoracoscopic surgery with anomalous vessel ligation and lingual segmentectomy were performed smoothly. The symptom of hemoptysis subsided after operation with 2-year follow up.

**Conclusion:**

An anomalous systemic arterial supply to the left upper lobe of the lung with an aberrant systemic system draining into the left pulmonary artery and co-supplying the lung parenchyma is extremely rare. Preoperative simulation with three-dimensional reconstruction image provides a clear spatial anatomy that allows clinicians to identify the orientation of the vessels more precisely when deciding on intervention.

## Background

Hemoptysis is one of the common symptoms in clinical practice. Besides the common causes of hemoptysis such as bronchiectasis, infections or malignancies, congenital vascular abnormalities are also potential differential diagnoses that are prone to be neglected [1]. Here we report an extremely rare case of a 70-year-old woman with an anomalous systemic artery draining into the pulmonary artery and co-supplying the left upper lobe of the lung. As three-dimensional (3-D) reconstruction image has gradually replaced the diagnostic role of angiography in the aspect of determination of anomalous vessels [2], we present this rare congenital scenario and emphasize the importance of 3-D reconstruction image in providing not only a diagnosis but also preoperative simulation.

## Case presentation

**Fig. 1:**
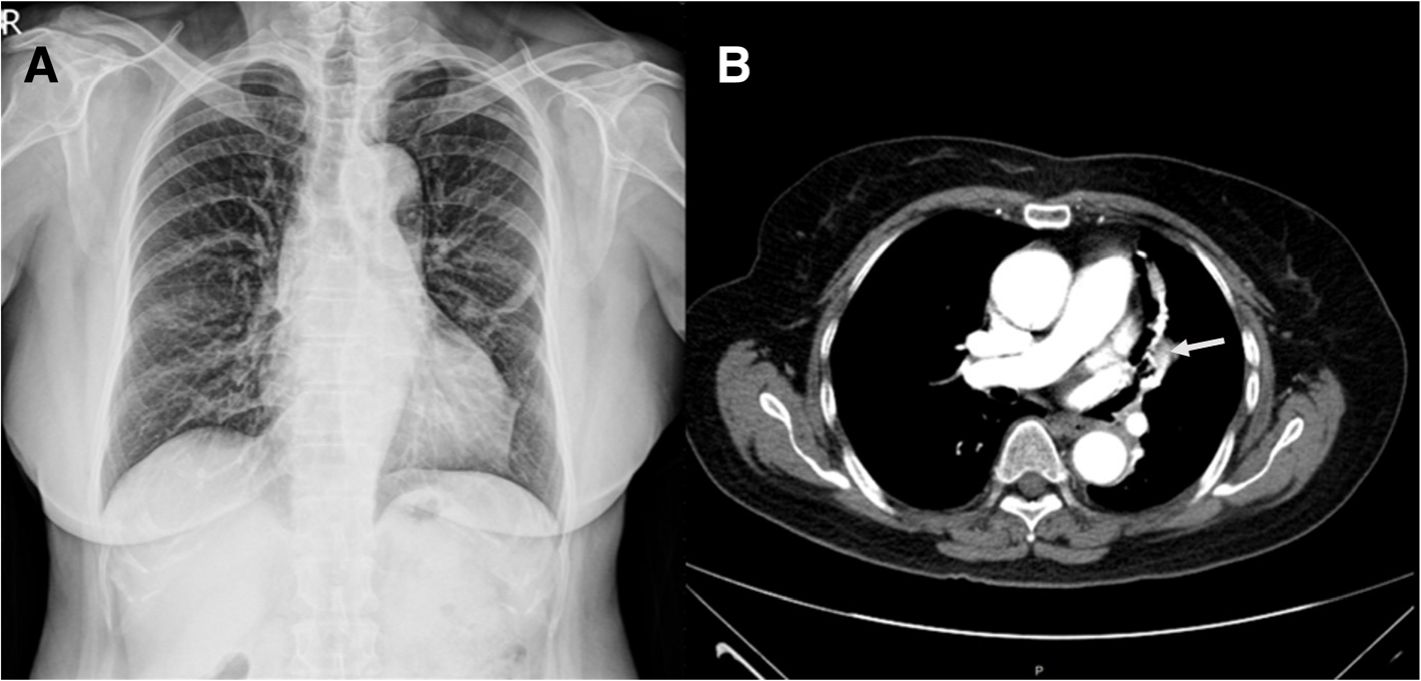
**a** A hyperdense nodular opacity is seen in the pericardial region in a plain X-ray film of the patient’s chest. **b** Computed tomography of chest with contrast illustrates a serpentine like structure (arrow) with avid enhancement in the lingula of the left lung abutting the pericardial region

**Fig. 2:**
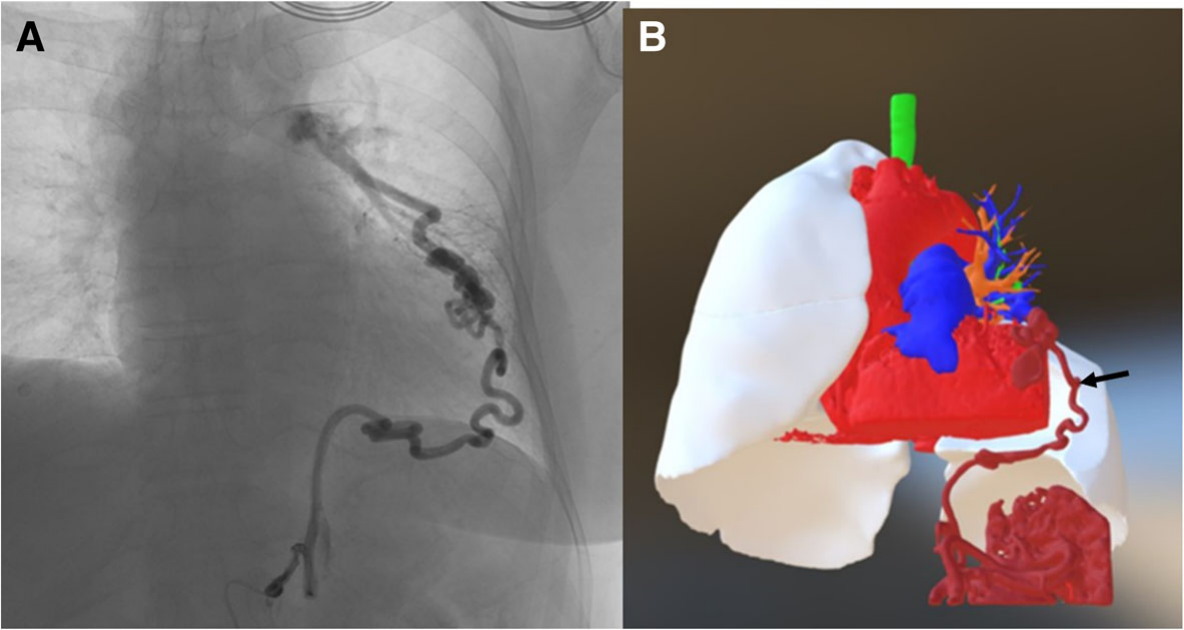
**a** Inferior phrenic artery catheterization angiography demonstrates the torturous engorged left inferior phrenic artery supplying (arrow) to vascular malformation of left lingular segment and communicating with left pulmonary artery. **b** The 3-D reconstruction image illustrates detailed spatial anatomy structure and orientation of the vessel

**Fig. 3:**
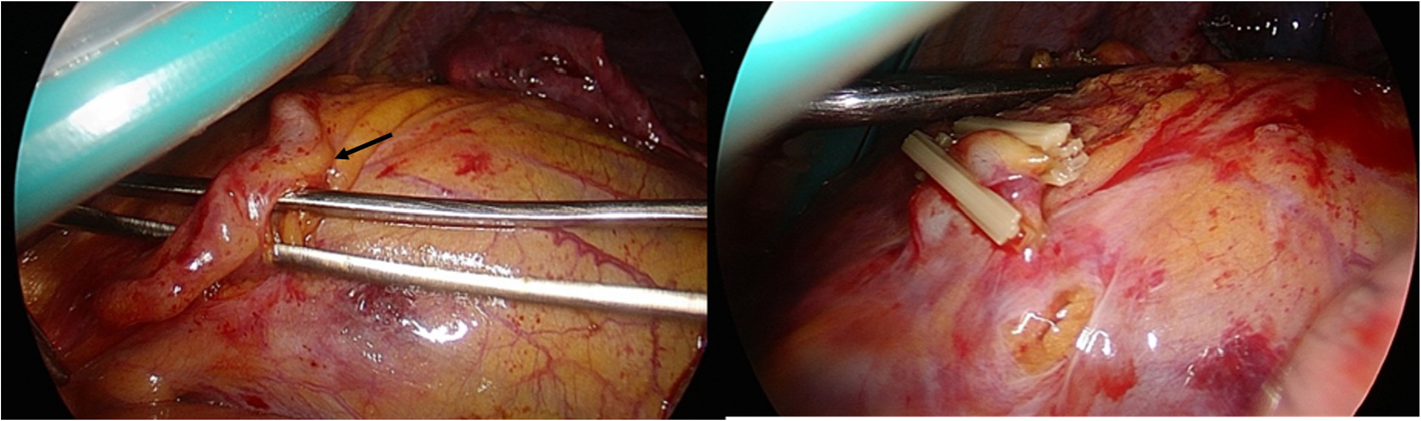
Intraoperative image of the aberrant vessel (arrow) abutting the pericardial region, which was then dissected and removed along with excision of lung parenchyma

The patient was a 70-year-old woman referred to our hospital because of four episodes of hemoptysis within one month. A history of myoma and pulmonary tuberculosis was noted before this admission. After admission, we arranged a series of examinations. No obviously abnormal findings were noted in the patient’s blood tests or sputum culture. Chest radiography revealed opacity of the left upper lung field (Fig. 1a). Chest multidetector computed tomography angiography (MDCTA) with 3-D volume rendering imaging demonstrated focal bronchiectasis and a 2.4 cm long serpentine hypervascular lesion in the lingula of the left lung abutting the pericardial region (Figs. 1b and 2b). Angiography revealed that the main supplying vessels of the hypervascular lesion arose from the inferior phrenic artery (Fig. 2a). The aberrant arterioles communicated with the inferior branch of the left pulmonary artery. Transcatheter arterial embolization (TAE) was attempted but failed because of the tortuosity of the vessels. Preoperative simulation with 3-D image reconstruction revealed the aberrant vessels and their associated anatomy. The patient underwent single-port video-assisted thoracoscopic surgery with segmentectomy of the lingula. Intraoperatively, the feeding artery of the serpentine hypervascular lesion was ligated and lingual segmentectomy was performed (Fig. 3). Histopathology of the resected specimens showed proliferative tortuous arterioles and vessels surrounded by lymphocytic aggregations. The patient was discharged on postoperative day 10 after an uncomplicated course. There was no hemoptysis with 2-year follow-up.

## Discussion

Hemoptysis is one of the most common symptoms in clinical practice. Bronchiectasis, tuberculosis, and malignancies account for most of the causes [1]. However, congenital vascular diseases, such as pulmonary arteriovenous malformations and arterial pulmonary malinosculation are also possible causes that are frequently neglected. Based on Pryce’s classification in 1946 [3], an anomalous systemic arterial supply without bronchopulmonary sequestration has been categorized as a Pryce type I abnormality. The most common aberrant arterial supply derives from the aorta and typically supplies the left lower lobe of the lung. Anomalous systemic vascular supply of the left upper lobe is especially rare and only four reported cases were found in a search of the literature [4–6]. Because of this anomalous anatomy, some issues such as secondary pulmonary hypertension and hemoptysis may be caused by connection of the systemic and pulmonary blood flows, which creates a left-to-left shunt. In our presenting case, a series of work up was performed initially to clarify the possible etiologies as the patient presented solely with hemoptysis. Due to negative findings of laboratory data and sputum cultures without obvious signs of infection, the imaging studies were arranged.

To identify aberrant vascular anatomy, multidetector computed tomography (MDCT) or multidetector computed tomography angiography (MDCTA) combined with 3-D reconstruction has gradually replaced the diagnostic role of angiography, which had been used conventionally for the determination of anomalous vessels. These new approaches are noninvasive and provide better spatial precision [2]. In our patient, both the image examination tools of MDCTA combined with 3-D volume rendering for reconstructing images and angiography were applied for a noninvasive approach to this vascular anomaly, aiming to delineate the anatomy of collateral aberrant vessels and planning surgical procedure [7], respectively, which provided a comparison between these two tools.

Lobectomy or segmentectomy, which aims at excision of the lung parenchyma perfused by the anomalous vessel, has been the standard treatment for this abnormality. Surgical ligation of the aberrant vessel has also been reported to be effective in eliminating symptoms [5]. However, TAE has been shown to be a promising alternative treatment for congenital arterial malformations. By placing endovascular devices, including metallic coils, vascular plugs, or other materials to occlude the aberrant artery, TAE provides a nonsurgical intervention choice that can be performed simultaneously while performing angiography. Although some severe complications including pulmonary infarction, and embolization of nontargeted arteries caused by migration of embolized materials, might be of concern, recurrent symptoms or serious complications have barely been reported [8, 9]. In our case, TAE was attempted while performing angiography but failed because of the tortuosity of the vessels. Surgical intervention with ligation of the hypervascular lesion and lingual segmentectomy were eventually performed without further complications or symptom remissions.

## Conclusion

An anomalous systemic arterial supply to the left upper lobe of the lung with an aberrant systemic system draining into the left pulmonary artery and co-supplying the lung parenchyma is extremely rare. Preoperative simulation with 3-D reconstruction images provides a clear spatial anatomy that allows clinicians to identify the orientation of the vessels more precisely when deciding on intervention.

## Abbreviations

3-D: Three dimensional
MDCT: Multidetector computed tomography
MDCTA: Multidetector computed tomography angiography
TAE: Transcatheter arterial embolization
